# Bile acid-receptor TGR5 deficiency worsens liver injury in alcohol-fed mice by inducing intestinal microbiota dysbiosis

**DOI:** 10.1016/j.jhepr.2021.100230

**Published:** 2021-01-19

**Authors:** Madeleine Spatz, Dragos Ciocan, Gregory Merlen, Dominique Rainteau, Lydie Humbert, Neuza Gomes-Rochette, Cindy Hugot, Nicolas Trainel, Françoise Mercier-Nomé, Séverine Domenichini, Virginie Puchois, Laura Wrzosek, Gladys Ferrere, Thierry Tordjmann, Gabriel Perlemuter, Anne-Marie Cassard

**Affiliations:** 1 Université Paris-Saclay, INSERM U996, Inflammation, Microbiome and Immunosurveillance, 92140, Clamart, France; 2 AP-HP, Hepatogastroenterology and Nutrition, Hôpital Antoine-Béclère, Clamart, France; 3 Université Paris-Saclay, Inserm U1193, Orsay, France; 4 UMR 7203, Laboratoire des Biomolécules, UPMC/CNRS/ENS, Paris, France; 5 Département PM2 Plateforme de Métabolomique, APHP, Hôpital Saint Antoine, Peptidomique et dosage de Médicaments, Paris, France; 6 Université Paris-Saclay, INSERM, CNRS, Institut Paris Saclay d’Innovation Thérapeutique, Châtenay-Malabry, France

**Keywords:** Alcoholic liver disease, Dysbiosis, Kupffer cells, Inflammation, Bile acid, Gut-liver axis, Microbiome, Alc, alcohol, ALD, alcohol-related liver diseases, alpha-SMA, alpha-smooth muscle actin, ALT, alanine aminotransferase, BA, bile acids, BHI, brain heart infusion, C57, conventional mice, C57^C57^, conventional mice transplanted with their own IM, CA, cholic acid, CCL, CC motif chemokine ligands, CDCA, chenodeoxycholic acid, Col1a1, collagen type-I alpha-1 chain, DCA, deoxycholic acid, FDR, false-discovery rate, FXR, farnesoid X receptor, IM, intestinal microbiota, KC, Kupffer cells, KO, knockout, LCA, lithocholic acid, LDA, linear discriminative analysis, LEfsE, LDA effect size, MCA, muricholic acid, mMMP9, matrix metallopeptidase 9, MO, monocytes/macrophages, NFkB, nuclear factor-kappa B, OTU, operational taxonomic unit, PCA, principal component analysis, PCoA, principal coordinate analysis, PICRUSt, phylogenetic investigation of communities by reconstruction of unobserved states, RIN, RNA integrity number, TBA, total bile acids, TG, triglycerides, TGF, transforming growth factor, TIMP1, tissue inhibitor of metalloproteinase 1, TNF, tumour necrosis factor, UDCA, ursodeoxycholic acid, WT, wild-type, WT^KO^, WT mice transplanted with the IM of TGR5-KO mice

## Abstract

**Background & Aims:**

Bile-acid metabolism and the intestinal microbiota are impaired in alcohol-related liver disease. Activation of the bile-acid receptor TGR5 (or GPBAR1) controls both biliary homeostasis and inflammatory processes. We examined the role of TGR5 in alcohol-induced liver injury in mice.

**Methods:**

We used TGR5-deficient (TGR5-KO) and wild-type (WT) female mice, fed alcohol or not, to study the involvement of liver macrophages, the intestinal microbiota (16S sequencing), and bile-acid profiles (high-performance liquid chromatography coupled to tandem mass spectrometry). Hepatic triglyceride accumulation and inflammatory processes were assessed in parallel.

**Results:**

TGR5 deficiency worsened liver injury, as shown by greater steatosis and inflammation than in WT mice. Isolation of liver macrophages from WT and TGR5-KO alcohol-fed mice showed that TGR5 deficiency did not increase the pro-inflammatory phenotype of liver macrophages but increased their recruitment to the liver. TGR5 deficiency induced dysbiosis, independently of alcohol intake, and transplantation of the TGR5-KO intestinal microbiota to WT mice was sufficient to worsen alcohol-induced liver inflammation. Secondary bile-acid levels were markedly lower in alcohol-fed TGR5-KO than normally fed WT and TGR5-KO mice. Consistent with these results, predictive analysis showed the abundance of bacterial genes involved in bile-acid transformation to be lower in alcohol-fed TGR5-KO than WT mice. This altered bile-acid profile may explain, in particular, why bile-acid synthesis was not repressed and inflammatory processes were exacerbated.

**Conclusions:**

A lack of TGR5 was associated with worsening of alcohol-induced liver injury, a phenotype mainly related to intestinal microbiota dysbiosis and an altered bile-acid profile, following the consumption of alcohol.

**Lay summary:**

Excessive chronic alcohol intake can induce liver disease. Bile acids are molecules produced by the liver and can modulate disease severity. We addressed the specific role of TGR5, a bile-acid receptor. We found that TGR5 deficiency worsened alcohol-induced liver injury and induced both intestinal microbiota dysbiosis and bile-acid pool remodelling. Our data suggest that both the intestinal microbiota and TGR5 may be targeted in the context of human alcohol-induced liver injury.

## Highlights

• TGR5 deficiency (TGR5-KO) worsened alcohol-induced liver injury in mice.
• This is linked to bile acid and intestinal microbiota changes.
• Bacterial genes involved in bile-acid transformation are decreased in TGR5-KO mice.
• Liver macrophage content increases without worsening of the inflammatory phenotype.
• Transferring TGR5-KO microbiota in wild-type mice exacerbates liver inflammation.

## Graphical abstract

**Figure:**
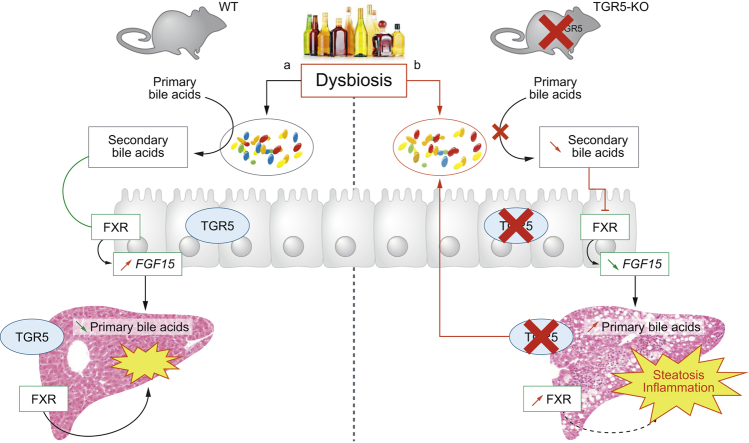

## Introduction

Alcohol-related liver disease (ALD) is a major cause of morbidity and mortality worldwide.^1^ It includes a broad spectrum of liver lesions, ranging from steatosis to inflammation, fibrosis, cirrhosis, and hepatocellular carcinoma.^2^ We have previously shown that the intestinal microbiota (IM) has a causal role in individual susceptibility to ALD, suggesting that a specific gut ecosystem can be protective or noxious. The analysis of intestinal metabolites in mice transplanted with the IM from alcoholic patients with severe liver injury shows specific features *vs.* those of mice transplanted with the IM from alcoholic patients without severe liver injury.^3^^,^^4^ The most discriminating molecules were bile acids (BAs).^3^ The amount of the primary BAs, chenodeoxycholic acid (CDCA), was higher in the faeces of mice receiving the deleterious human IM, whereas the secondary BA, ursodeoxycholic acid (UDCA), was more abundant in mice that received the protective IM. Primary BAs are produced in hepatocytes from cholesterol, conjugated to glycine (mainly in humans) or taurine (mainly in rodents). Secreted in bile, they reach the gut lumen and, once in the ileum, are mostly reabsorbed and transported back to the liver through the so-called enterohepatic cycle.^5^ The non-absorbed primary BAs remaining in the gut are transformed by gut bacteria into more hydrophobic secondary BAs, which are passively reabsorbed in the colon, with only minor faecal BA loss. As a consequence, the IM (and eventual dysbiosis) has a major impact on the composition of the BA pool and thus on BA signalling through their receptors.^6^ Although the historical function of BAs is to facilitate the absorption of dietary lipids and lipid-soluble nutrients,^7^ they are now considered to be signalling molecules that act through the activation of receptors, mainly the nuclear farnesoid X receptor (FXR) or TGR5 (also known as GPBAR1).[8], [9], [10] BAs modulate energy metabolism through FXR activation in the gut and liver, as well as their own biosynthesis, by a negative feedback loop following FXR activation.^11^ The role of FXR in several liver diseases has already been studied and it has been shown that FXR deficiency worsens liver injury in alcohol-fed mice.^12^^,^^13^ Conversely, ileum FXR activation has a protective effect.^14^ Far less studied than FXR for its properties in the liver, TGR5 is involved in a variety of functions, including metabolic expenditure, the inflammatory response, gut motility, and gallbladder homeostasis.[15], [16], [17] Moreover, TGR5 activation in the monocyte/macrophage (MO) lineage improves liver injury through antagonism of NFκB in Kupffer cells (KCs)^18^ and the suppression of pro-inflammatory cytokine production and phagocytic functions in MOs.^19^^,^^20^ TGR5 activation is also reported to be hepatoprotective in the context of BA overload, liver regeneration, and the setting of cholestasis^17^^,^^21^ through yet incompletely explored mechanisms. Among them, a TGR5-dependent anti-inflammatory mechanism and the regulation of epithelial permeability have been proposed.^22^ Whether there is an impact on the composition of the BA pool and/or the IM is still debated.^17^ TGR5 has been little explored in the context of ALD, although it was recently suggested that a TGR5 agonist may improve the disease in mice.^23^

Here, we addressed the role of TGR5 and its interaction with the IM in liver lesions induced by alcohol consumption in mice. We provide evidence that the absence of TGR5 is associated with the worsening of liver steatosis and inflammation. Upon alcohol consumption, we observed dysbiosis of the IM and a related profound alteration of the composition of the BA pool, with an impact on the regulation of BA synthesis, steatosis, and inflammatory processes.

## Materials and methods

### Mice

Female TGR5-knockout (TGR5-KO) and wild-type (WT) mice were kindly provided by T. Tordjmann, in agreement with Merck Research Laboratories (Kenilworth, NJ, USA). These mice were generated in a hybrid (129S3/SvImJ×C57BL/6) background^23^ and included in the protocol at between 7 and 10 weeks old. Seven-week-old female C57BL/6J mice were purchased from Janvier laboratory (Le Genest, France).^24^ Animals were kept in humidity- and temperature-controlled rooms on a 12-h light–dark cycle and had access to a chow diet and water *ad libitum* before the study. The animal experimentation procedure was validated by the French Ministry, APAFIS 9600-2017041417257524 v3.

Microbiota transfer was performed by faeces gavage using a modified version of a previously described protocol^25^ as follows: Faeces were recovered from 10 mice (C57BL/6J or TGR5-KO mice), diluted in BHI (Brain Heart Infusion, Becton Dickinson) supplemented with 0.5 mg/ml L-cysteine (Sigma-Aldrich, St Louis, MO, USA) and 20% skim milk (Becton Dickinson) (vol/vol) and stored in aliquots at -80°C.

For the microbiota transfer experiments, 100 μl containing 3.33 mg of faeces was administered to each corresponding mouse 2 times per week for 21 days by oral gavage.

### Chronic exposure to ethanol

Seven- or 10-week-old mice were fed a liquid diet adapted from Lieber DeCarli for 21 days based on the NIAAA model^26^ and previously described.^27^ Briefly, the ethanol diet was obtained by adding absolute ethanol to a solution of Lieber DeCarli powder (Ssniff, Spezialdiäten GmbH, Soest, Germany) in filtered water. After a 7-day period of adaptation to the animal facility and a 7-day period of adaptation to the semiliquid diet, mice were given increasing amounts of ethanol for 7 days (1% increase every 2 days). The final concentration of ethanol in the liquid diet was 5% (vol/vol), such that ethanol accounted for 28% of the total caloric intake. The control diet was obtained by replacing the ethanol with an isocaloric amount of maltodextrin (Maldex 150, Safe, France). The alcohol-fed groups were allowed free access to the 5% (vol/vol) ethanol diet for 7 days. Control mice were fed the isocaloric control diet throughout the feeding period. Body weight and food intake were measured once every 2 days.

### Tissues and samples

Mice were anaesthetised and blood samples collected in EDTA-coated tubes. The serum was used for liver alanine aminotransferase (ALT) determination and bile-acid measurement. The livers were excised, weighed, and either fixed in 4% buffered paraformaldehyde or frozen for further triglyceride (TG) and bile-acid measurement and RNA extraction. The proximal ileum and colon were cut into 2 pieces: 1 was flushed, longitudinally opened, cut into 2-cm sections, and fixed in 4% paraformaldehyde and the other was frozen for RNA extraction. The caecal content was collected and frozen for caecal bile-acid measurement. Faecal samples were collected from mice 2 days before euthanasia for gut microbiota analysis. All samples were stored at -80°C until use.

### Isolation of liver MOs

To recover liver MOs, the livers were perfused inversely to the normal flux with PBS/EDTA (5 mM). After removing the blood, livers were excised and homogenised with 0.05% collagenase IV (Sigma–Aldrich, Saint-Louis, MO, USA) buffered with 0.1 M HEPES for 20 min at 37°C. Hepatocytes were removed by a short centrifugation at 50xg. The non-parenchymatous cells were filtered through a 70-μm filter and resuspended with 22% Optiprep (Axis-Shield) for liver MO enrichment, layered with HBSS/EDTA (5 mM) and centrifuged at 900xg at room temperature for 20 min. Cell viability was assessed by trypan-blue labelling, as previously described.^28^

### Measurement of liver TGs and plasma transaminases

TGs were extracted using an Abcam Triglyceride Assay Kit - Quantification (Cambridge, UK) and measured with a Mithras LB940 (Berthold Technologies). The level of TG is expressed in nmol per milligram of liver. Transaminases (ALT and aspartate transaminase) were assessed by a spectrophotometric method (Olympus, AU400).

### Liver and gut histology

The liver and gut (ileum, colon) were fixed overnight in 4% paraformaldehyde and embedded in paraffin. Liver paraffin sections (3 μm thick) were stained with hematoxylin and eosin (H&E) or Picrosirius Red. A sample of liver was frozen in tissue-freezing medium (Microm-microtech). Frozen sections (7 μm thick) were used for Oil Red O staining using standard procedures. For Oil Red O staining, area measurement was performed using the ImageJ software (https://imageJ.nih.gov). We used ‘Thresholding’ to define the region of interest (staining or fluorescence) and the ‘Freehand selection’ tool to determine the total area of interest. Immunofluorescence or immunohistochemistry staining for F4/80 was performed on 3-μm sections of paraffin-embedded livers from WT and KO alcohol-fed mice. The paraffin was removed and the sections rehydrated. Sections were then stained by immunofluorescence using a mAb against F4/80 (Bio-Rad, France) at a concentration of 10 μg/ml overnight, washed, and incubated with a secondary antibody Alexa Fluor 594 (Thermo Fischer, France) for 45 min at room temperature or washed and incubated with a biotinylated secondary antibody and then with a streptavidin–horseradish peroxidase complex (LSAB kit, Dako) and counterstained with haematoxylin for immunohistochemistry.

Colon paraffin sections (3 μm thick) were labelled with antibodies purchased from Abcam against ZO-1 (ab96587) and Occludin (ab216327), followed by staining with a fluorochrome-coupled secondary antibody goat anti-rabbit Alexa FluorTM Plus 594 (Invitrogen, Thermo Fisher Scientific). Nuclei were stained with Hoechst (Molecular probes).

Slides were scanned using NanoZoomer 2.0-RS digital slide scanner (Hamamatsu, Japan). Images were digitally captured from the scanned slides using NDP.view2 software (Hamamatsu).

### RNA extraction and quantification

Livers were disrupted in Qiazol solution and total RNA extracted using a Qiagen RNeasy Lipid Tissue Mini Kit (Courtaboeuf, France). Total gut RNA was extracted using a Qiagen RNeasy Plus Mini Kit. Both were extracted after being disrupted with an MP Biomedicals FastPrep. Liver MO RNA was extracted using a Qiagen RNeasy Plus Mini Kit. The RNA integrity number (RIN) was determined using an Agilent Bioanalyzer 2100 system with the RNA 6000 Nano Labchip kit. Samples had a RIN of 8 for liver tissues and 7 for isolated liver MO and gut tissues. For cDNA synthesis, 1 μg of each total RNA sample was reverse transcribed. A 12-μl mix containing 1 μg RNA, random hexamers (Roche Diagnostics, Meylan, France), and 10 mM dNTP Mix (Invitrogen, Carlsbad, CA, USA) was prepared for each sample. Mixtures were heated at 65°C for 5 min, cooled on ice, and then an 8-μl reaction mix containing 1 μl M-MuLv RT (Invitrogen), 4 μl 5x buffer (Invitrogen), 2 μl 0.1 M dithiothreitol (Invitrogen), and 1 μl Protector RNase Inhibitor (40 U/μl; Invitrogen) was added. The reaction conditions were 10 min at 25°C, 50 min at 50°C, and 15 min at 70°C.

### Gene expression analysis by quantitative PCR

Real-time qPCR was performed in a Light Cycler 480 (Roche Diagnostics) using the LC FastStart DNA Master SYBR Green I kit (Roche Diagnostics). Amplification was initiated with an enzyme activation step at 95°C for 10 min, followed by 40 cycles, consisting of a 20-s denaturation step at 95°C, a 15-s annealing step at the temperature appropriate for each primer, and a 10-s elongation step at 72°C. The Primer sequences of the amplified targets are listed in Table S1. Data were analysed using LC 480 Software (Roche Diagnostics). Arbitrary units represented the ratio between the gene expression of the target and the gene expression of a housekeeping gene used as a reference gene. We used the 18S and glyceraldehyde 3-phosphate dehydrogenase (GAPDH) as the reference gene.

### Measurement of BAs

Concentrations of BA molecular species were measured by HPLC coupled to tandem mass spectrometry (HPLC-MS/MS) as previously described.^29^ Standard stock solutions were prepared in methanol at a concentration of 1 mg/ml and stored in a sealed container at -20°C. The stock solutions were pooled and diluted to obtain mixed calibration BA solutions. Standard solutions were available to quantify BA.

### Bacterial DNA extraction and analysis of the gut microbiota by 16S ribosomal RNA sequencing

Bacterial DNA was extracted from faeces using a Qiagen QIAamp DNA Stool Mini Kit, after being disrupted with an MP Biomedicals FastPrep. The composition of the faecal microbiota was analysed using Illumina MiSeq technology targeting the 16S ribosomal DNA V3-V4 region in paired-end modus (2x300 base pairs; GenoToul, Toulouse, France) as described previously.^27^

The non-chimeric sequences were then clustered into operational taxonomic units (OTUs) at 97.0% sequence similarity using a closed reference-based picking approach with UCLUST software against the Greengenes database 13_8 of bacterial 16S rDNA sequences.^30^ The mean number of quality-controlled reads was 18,302 ± 7,625 (mean ± SD) per mouse. After rarefaction at 4,000 reads per sample, bacterial alpha diversity was estimated using the Shannon Index. OTUs with a prevalence <5% were removed from the analysis. Analyses using R software v2.14.1 were restricted to merged OTUs with the same taxonomic assignment. Results are represented as the mean ± SEM. The Wilcoxon test was used to assess statistical significance of the bacterial composition between the various samples. Associations were considered to be significant after a false-discovery rate (FDR) correction of the *p* value (*q* <0.05).

Beta diversity was assessed using weighted and unweighted UniFrac distances. The weighted Unifrac metric is weighted by the difference in the abundance of OTUs from each community, whereas unweighted UniFrac only considers the presence/absence of the OTUs providing different information. Both are phylogenetic beta diversity metrics. The link between the various groups of mice and bacterial microbial profiles was assessed by performing an ANOSIM test with 10,000 permutations on the beta diversity metrics described above. Linear discriminative analysis (LDA) effect-size (LEfSe) analysis was performed to identify the taxa displaying the largest differences in abundance in the microbiota between groups.^31^ Only taxa with an LDA score >2 and a significance of α <0.05, as determined with Wilcoxon signed-rank tests, are shown.

The functional composition of the intestinal metagenome was predicted using Phylogenetic Investigation of Communities by Reconstruction of Unobserved States (PICRUSt).^32^ LEfSe and PICRUSt were accessed online (http://huttenhower.sph.harvard.edu/galaxy/).

### Statistical analyses

Results are shown as mean ± SEM. The non-parametric Kruskall-Wallis test and Dunn’s multiple comparison post test were used to compare the means of groups as appropriate (Graphpad Prism 7.0a, Graphpad Software Inc, La Jolla, CA, USA); *p* <0.05 was considered to be statistically significant: ∗*p* <0.05, ∗∗*p* <0.01, ∗∗∗*p* <0.001.

## Results

### TGR5 deficiency worsens alcohol-induced liver injury

**Fig. 1:**
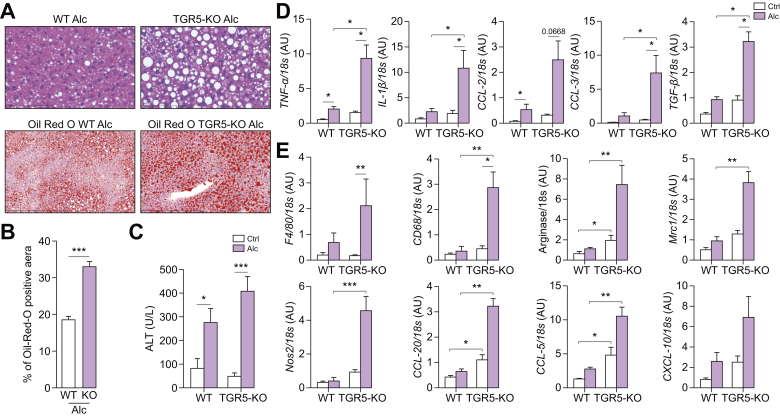
**Liver injury in WT and TGR5-deficient mice after chronic alcohol consumption.** Wild-type (WT) and Takeda-G-protein-receptor-5-deficient (TGR5-KO) mice were fed alcohol (Alc) or isocaloric maltodextrin (Ctrl). (A) Representative histological images of H&E and Oil Red O staining of the liver of alcohol-fed mice (scale bar: H&E = 100 μm and Oil Red O = 400 μm). (B) Quantification of Oil Red O staining. (C) Plasma ALT levels. (D–E) Liver mRNA levels of (D) pro-inflammatory cytokines and chemokines and (E) genes related to the macrophage phenotype. Kruskall-Wallis test and Dunn’s multiple comparison post-hoc test, ∗*p* <0.05, ∗∗*p* <0.01, ∗∗∗*p* <0.001. WT Ctrl n = 4, WT Alc n = 6, TGR5-KO Ctrl n = 4, TGR5-KO Alc n = 6. ALT, alanine aminotransferase.

We investigated the role of TGR5 in the involvement of alcohol-induced liver injury by feeding alcohol to TGR5-KO and control WT mice. Liver injury was significantly worse in alcohol-fed TGR5-KO than WT mice, although alcohol consumption was similar (Fig. S1A). TGR5-KO alcohol-fed mice showed increased steatosis, as shown by H&E and Oil Red O staining of liver sections and quantification of Oil Red O staining (Fig. 1A and B). Accordingly, lipogenesis and mRNA levels of TG synthesis enzymes were higher in TGR5-KO than WT mice (Fig. S1B and C). Alcohol-fed TGR5-KO mice also showed higher ALT levels and higher mRNA levels of liver pro-inflammatory cytokines, chemokines, and activated MO markers than WT mice (Fig. 1C–E). Of note, the increase in TGF-β levels is in accordance with the increase in the expression of fibrosis-associated genes and fibrotic scars (Fig. S2A and B). There was no increase in ileal inflammation between WT and TGR5-KO mice, independently of alcohol intake (Fig. S3A). However, we found that alcohol induced changes in intestinal permeability, as shown by decreased expression of ZO-1 and occludin, whereas there was no difference in their expression depending on the genotype of the mice (Fig. S3B and C).

Liver inflammation was associated with a higher number of liver MOs in alcohol-fed TGR5-KO than WT mice, as shown by higher F4/80 mRNA levels (Fig. 1E), as well as greater F4/80-positive immunohistochemistry staining (Fig. S4A). However, isolated MOs from WT and TGR5-KO alcohol-fed mice were not significantly different in terms of pro-inflammatory cytokine mRNA expression profiles (Fig. S4B), suggesting that the *in vivo* phenotype observed in the absence of TGR5 cannot be solely explained by an intrinsic exacerbated inflammatory MO profile. Nevertheless, the increase in CC motif chemokine ligand-2 (CCL-2) and CXC motif chemokine ligand-10 (CXCL-10) mRNA levels likely correlate with higher MO recruitment to the liver (Fig. S4B). Of note, alcohol did not induce any significant increase in TGR5 mRNA levels in the liver MOs of WT mice (Fig. S4C). Thus, our data show that the lack of TGR5 was associated with the worsening of liver steatosis and inflammation, but that MOs, although more highly recruited to the livers of TGR5-KO mice, cannot be considered to be primarily responsible for this phenotype.

### The lack of TGR5 is associated with an altered IM in mice fed a normal or alcohol-enriched diet

**Fig. 2:**
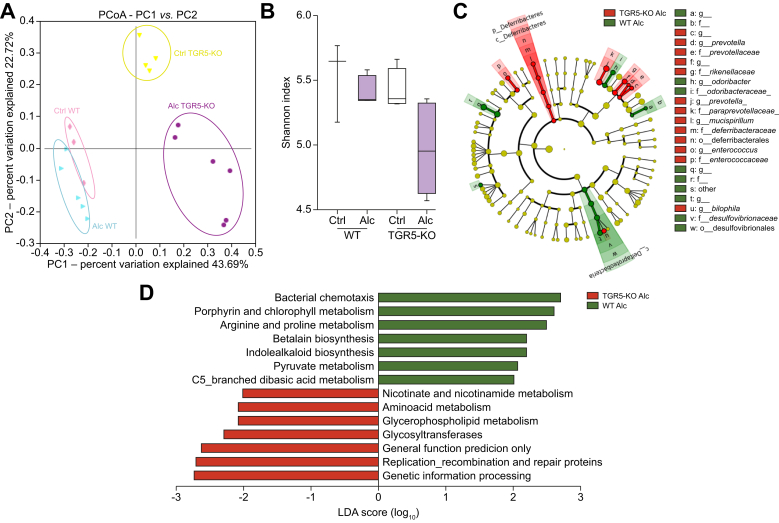
**Intestinal microbiota of control and alcohol-fed WT and TGR5-deficient mice**. WT and TGR5-KO mice were fed alcohol (Alc) or isocaloric maltodextrin (Ctrl). (A) Bray Curtis PCoA Unifrac distances showing a difference in the composition of the faecal microbiota between groups (*p* = 0.001). (B) Box plots showing alpha diversity based on the Shannon Index (Kruskall-Wallis test and Dunn’s multiple comparison post-hoc test). (C) Cladogram showing taxa with the largest differences (LDA >2) in abundance by LEfSe analysis. (D) LEfSe for the predicted metagenome metabolic pathways (KEGG modules). Only taxa with an LDA >2 and *p* <0.05 are shown (Wilcoxon test). WT Ctrl n = 3, WT Alc n = 5, TGR5-KO Ctrl n = 4, TGR5-KO Alc n = 6. LDA, linear discriminative analysis; LEfSe, linear discriminative analysis effect size; PCoA, principal coordinate analysis; WT, wild-type.

The IM has been identified as an important player in the pathophysiology of ALD and individual susceptibility to alcohol toxicity. We considered the possibility that the lack of TGR5 is associated with an alteration of the gut microbiota of alcohol-fed mice, which in turn may result in the worsening of liver injury. Faecal 16S sequencing showed that alcohol modified the IM of both WT and TGR5-KO mice. Strikingly, TGR5 deficiency itself induced specific dysbiosis (Fig. 2A), without differences between groups based on alpha-diversity (Fig. 2B). Linear discriminant analysis (LEfSe) showed significant differences in several taxonomic ranks (Fig. 2C), including an increase in the Deferribacteres phylum and the *Mucispirillum, Enterococcus, Prevotella*, and *Bilophila* genera between alcohol-fed TGR5-KO and WT mice. We explored the biological impact of the observed dysbiosis in alcohol-fed TGR5-KO mice by generating the predicted metagenome using PICRUSt, which yielded 328 pathways. Among the pathways significantly enriched in alcohol-fed TGR5-KO mice were those involved in lipid, amino-acid, cofactor, and vitamin metabolism (Fig. 2D). These results suggest that TGR5 deficiency induces specific dysbiosis, with a shift in metabolic functions, independently of alcohol administration, and that such dysbiosis could be responsible for the worsening of alcohol-induced liver injuries in TGR5-KO mice.

### The IM of TGR5-KO mice is associated with alcohol-induced liver injury

**Fig. 3:**
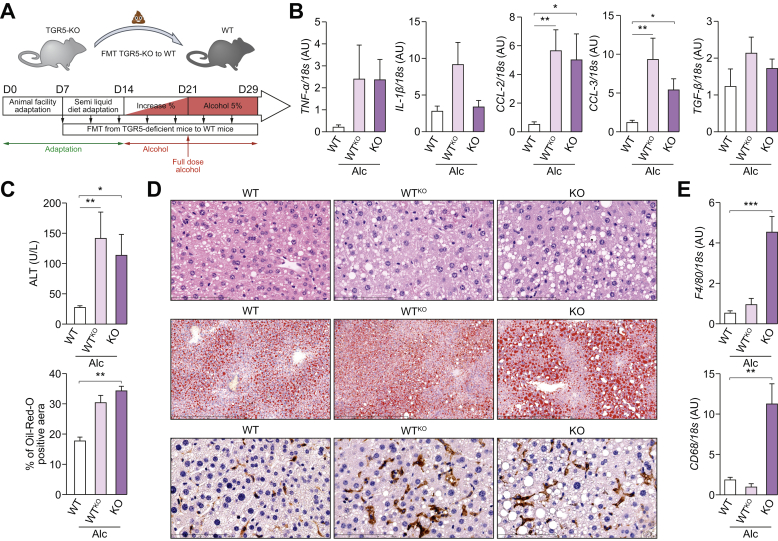
**Faecal microbiota transplantation from TGR5-KO to WT mice worsens alcohol-induced liver injury**. (A) Experimental design. (B–D) Wild-type (WT), Takeda-G-protein-receptor-5-deficient (TGR5-KO), and WT mice transplanted with the IM of TGR5-KO mice (WT^KO^) were fed alcohol. (B) Liver mRNA levels of pro-inflammatory cytokines and chemokines. (C) Plasma ALT and quantification of Oil Red O staining. (D) Representative histological images of the liver (upper, scale bar = 100 μm), Oil Red O staining (middle, scale bar = 400 μm), and F4/80 labelling (lower, scale bar = 100 μm). (E) Liver mRNA expression of *F4/80* and *CD68*. Kruskall-Wallis test and Dunn’s multiple comparison post-hoc test, ∗*p* <0.05, ∗∗*p* <0.01, ∗∗∗*p* <0.001. WT n = 5, WT^KO^ n = 5, KO n = 9. IM, intestinal microbiota.

**Fig. 4:**
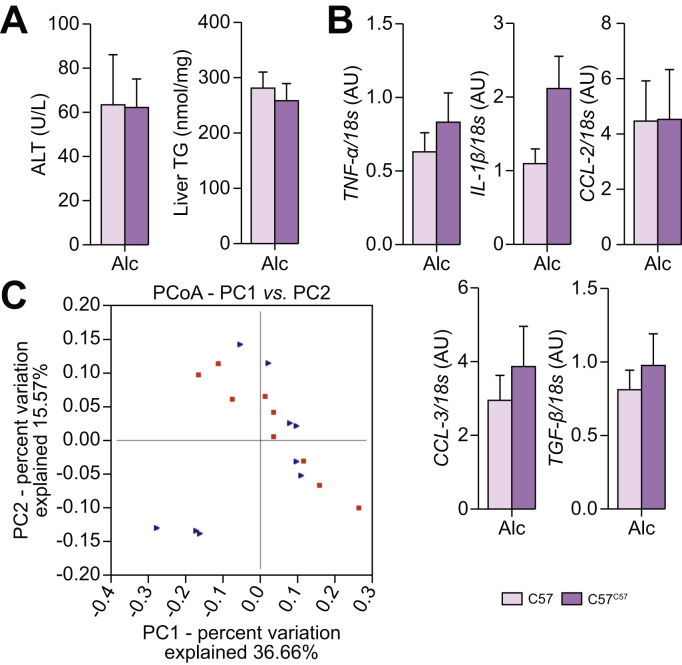
**Liver injury in alcohol-fed mice transplanted with their own IM and analysis of the IM**. C57BL/6J (C57) mice were fed alcohol. A group was transplanted with their own IM (C57^C57^). (A) Plasma ALT and liver TG levels. (B) Liver mRNA levels of pro-inflammatory cytokines and chemokines. (C) Bray Curtis PCoA Unifrac distances showing no difference in the composition of the faecal microbiota between groups. Mann-Whitney test. C57 n = 9, C57^C57^ n = 9. ALT, alanine aminotransferase; IM, intestinal microbiota; PCoA, principal coordinate analysis; TG, triglycerides.

We next examined the role of the dysbiosis induced by the absence of TGR5 expression in the worsening of alcohol-induced liver injury by transplanting the IM of TGR5-KO mice into conventional WT mice before feeding them alcohol (Fig. 3A). After alcohol feeding, WT mice receiving the TGR5-KO IM (WT^KO^) showed similar worsening of liver inflammation as TGR5-KO mice. Indeed, plasma ALT and liver TNFα, CCL-2, and CCL-3 mRNA levels (Fig. 3B and C) were higher in alcohol-fed TGR5-KO and WT^KO^ mice than in alcohol-fed WT mice. However, although we observed greater steatosis in alcohol-fed WT^KO^ by histological analysis, quantification of the Oil-Red-O-positive area between WT^KO^ and WT or TGR5-KO mice showed no statistically significant difference (Fig. 3C and D). In addition, although we observed higher levels of liver CCL-2 mRNA in WT^KO^ mice, they did not show significantly greater liver MO recruitment (Fig. 3D, lower panel, and E). This could have been expected, as TGR5 is reported to reduce MO migration and the MOs from WT^KO^ mice obviously still expressed TGR5.^33^ The worsening of liver injury by IM transplantation was specific to the TGR5-KO IM. Indeed, we excluded that repeated force-feeding of the IM had an effect *per se* on liver injury by comparing the liver injury of alcohol-fed C57BL6/J mice (C57) and that of C57BL6/J mice transplanted with the IM of a pool of C57BL6/J faeces (C57^C57^). There was no difference between mice, either in terms of liver lesions after consuming alcohol or intestinal dysbiosis (Fig. 4).

**Fig. 5:**
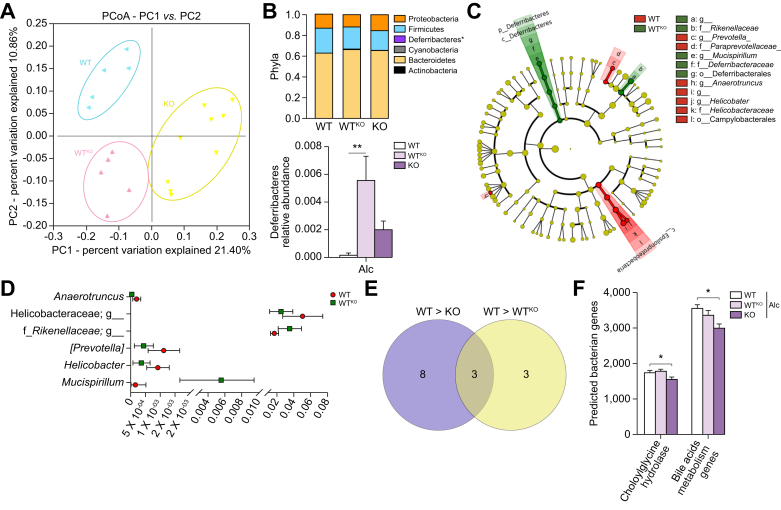
**Intestinal microbiota of alcohol-fed mice transplanted with the IM of TGR5-deficient mice**. WT, KO, and WT^KO^ mice were fed alcohol. (A) Unweighted PCoA Unifrac distances showing the composition of the IM (ANOSIM test, *p* = 0.001). (B) Histogram showing the relative abundance between phyla (FDR test, *p* <0.05) (upper panel) and Deferribacteres (FDR test, *p* <0.05) (lower panel). (C–E) Comparisons between WT and WT^KO^ mice. (C) Cladogram showing taxa with the largest differences in abundance (LDA >2). (D) Plot showing differences in the relative abundance of taxa. (E) Venn diagram showing common taxa. (F) Predictive expression of choloylglycine hydrolase and BA metabolism genes. Kruskall-Wallis test and Dunn’s multiple comparison post-hoc test, ∗*p* <0.05. WT n = 5, WT^KO^ n = 5, KO n = 9. BA, bile acid; IM, intestinal microbiota; KO, knockout; LDA, linear discriminative analysis; PCoA, principal coordinate analysis; WT, wild-type.

The IM of alcohol-fed WT^KO^ mice was modified and shared a similar increase in Deferribacteres with the IM of TGR5-KO mice (Fig. 5A–C). LEfSe identified several taxa modified in these mice, including an increase in the relative abundance of *Mucispirillum* among Deferribacteres (Fig. 5D). We also observed an increase in the relative abundance of an unidentified taxon of the *Rikenellaceae* family and a decrease in that of *Prevotella* and *Anaerotruncus*, as well as *Helicobacter* and *Campylobacterales*. We compared the differences between WT and WT^KO^ mice to those observed between WT and TGR5-KO mice to identify candidate taxa related to the worsening of alcohol-induced liver injury in TGR5-KO mice. Only 3 taxa were common between the 2 comparisons (Fig. 5E, mixed yellow and purple): (1) *Mucispirillum,* belonging to Deferribacteres, and harbouring a unique *Mucispirillum schaedleri species;* (2) the unidentified taxa from the *Rikenellaceae* family, represented by 2 species of the *Alistipes* genus*: Alistipes putredinis* and *Alistipes finegoldii;* and (3) Paraprevotella. In terms of predicted metagenomic functions, the abundance of the bacterial genes involved in primary BA transformation into secondary BAs (cholylglycine hydrolase [EC:3.5.1.24], 7-alpha-hydroxysteroid dehydrogenase [EC:1.1.1.159], and 3-dehydro-bile acid delta4,6-reductase [EC:1.3.1.114]), was lower in alcohol-fed TGR5-KO than WT mice (Fig. 5F).

## BA pool composition and BA synthesis are altered in TGR5-KO mice upon alcohol feeding

**Fig. 6:**
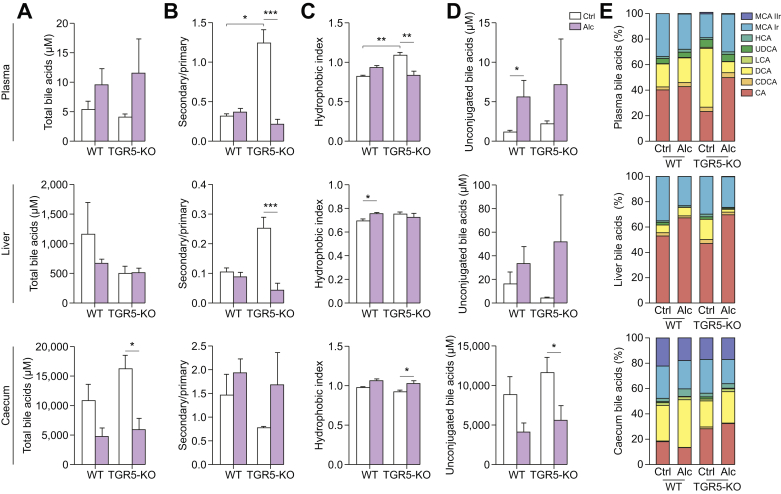
**Bile acids in alcohol-fed WT and TGR5-deficient mice**. WT and TGR5-KO mice were fed alcohol (Alc) or isocaloric maltodextrin (Ctrl). Bile-acid composition in the plasma (upper), liver (middle), and caecum (lower). (A) Total bile acids. (B) Ratio of secondary/primary bile acids. (C) Hydrophobic index. (D) Unconjugated bile acids. (E) Percentage of each bile acid. Kruskall-Wallis test and Dunn’s multiple comparison post-hoc test, ∗*p* <0.05, ∗∗*p* <0.01, ∗∗∗*p* <0.001. WT Ctrl n = 4, WT Alc n = 6, TGR5-KO Ctrl n = 4, TGR5-KO Alc n = 6. CA, cholic acid; CDCA, chenodeoxycholic acid; DCA, deoxycholic acid; HCA, hyocholic acid; LCA, lithocholic acid; MCA, muricholic acid; WT, wild-type.

On the basis of the data reported above, we next explored the impact of TGR5 on BA content in various compartments (plasma, liver, and caecum) in the different groups of mice given a normal or alcohol-enriched diet. We first observed that there was no significant overload of total BAs (TBAs) in alcohol-fed mice *vs.* those fed a normal diet, regardless of the genotype, as shown by the absence of an increase in TBA concentration in the plasma and liver (Fig. 6A). Conversely, there was a decrease in the TBA concentration in the caecum, independently of the genotype. Interestingly, BA composition was highly modified in TGR5-KO mice upon alcohol consumption relative to those given a normal diet. TGR5-KO mice fed a normal diet showed higher plasma and liver concentrations of secondary BAs (Fig. 6B), as well as a higher hydrophobic index (Fig. 6C), than WT mice, consistent with a more hydrophobic BA pool, as already reported for these mice.^21^^,^^24^ However, alcohol consumption abolished these discrepancies. Indeed, upon alcohol consumption, the TGR5-KO mice showed a dramatic decrease in secondary BA levels, especially DCA, with a concomitant reduction in the hydrophobicity index of the BA pool in plasma (Fig. 6C and E), whereas alcohol-fed WT mice did not (Fig. 6B and D). As already reported,^14^ the level of unconjugated BAs was higher in alcohol-fed mice, regardless of the genotype (Fig. 6D). There was no difference in the overall BA composition between the plasma and caecum of alcohol-fed WT, WT^KO^, and KO mice (Fig. S5). However, in the liver, we observed a higher secondary to primary BA ratio in alcohol-fed WT^KO^ than alcohol-fed WT and KO mice, as well as a higher hydrophobicity index in alcohol-fed WT^KO^ than alcohol-fed WT mice. These data suggest that transfer of the IM from TGR5-KO to WT mice was followed by modifications in liver BA composition resembling those observed in TGR5-KO mice.

**Fig. 7:**
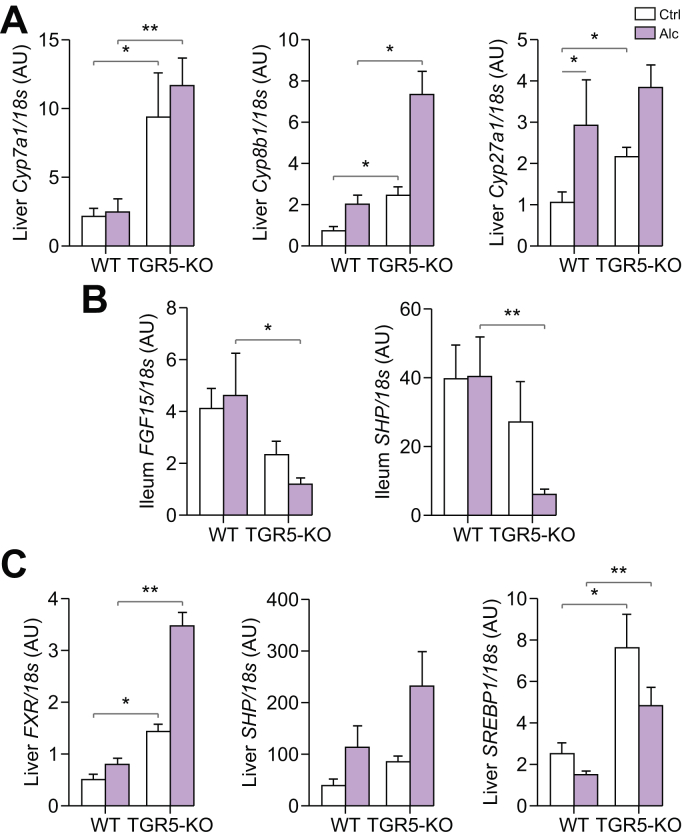
**Quantification of mRNA levels for genes involved in bile-acid synthesis**. WT and KO mice were fed alcohol (Alc) or isocaloric maltodextrin (Ctrl). (A–C) mRNA level of genes quantified by qPCR related to (A) hepatic bile-acid synthesis enzymes (*Cyp7a1*, *Cyp8b1*, *Cyp27a1*) and the FGF15 pathway in the (B) ileum (*FGF15*, *SHP*) and (C) liver FXR, SHP and SREBP1. Kruskall-Wallis test and Dunn’s multiple comparison post-hoc test, ∗*p* <0.05, ∗∗*p* <0.01. WT Ctrl n = 4, WT Alc n = 6, TGR5-KO Ctrl n = 4, TGR5-KO Alc n = 6. FGF15, fibroblast growth factor-15; KO, knockout; WT, wild-type.

DCA is an FXR agonist involved, in particular, in the negative feedback of BA synthesis.^34^ Thus, we examined liver CYP mRNA expression and that of the ileal FXR signalling pathway. The levels of liver mRNA coding for CYP7a1, the limiting enzyme of BA synthesis, as well as that for CYP8b1, were markedly higher in alcohol-fed TGR5-KO than WT mice (Fig. 7A), in agreement with activation of the classical pathway of BA synthesis and the related increase in CA in alcohol-fed TGR5-KO mice (Fig. 6E). These data suggest that the lack of TGR5 results in the absence of feedback inhibition on BA synthesis upon alcohol consumption. Accordingly, there was also a significant decrease in ileal SHP mRNA levels, suggesting downregulation of the fibroblast growth factor-15 pathway in alcohol-fed TGR5-KO mice (Fig. 7B). Moreover, we found significant compensatory overexpression of FXR mRNA in the liver of TGR5-KO mice (Fig. 7C). This was associated with higher hepatic expression of the FXR target genes SHP and SREBP1 (Fig. 7C), involved in lipid metabolism, and correlated with the greater steatosis in alcohol-fed TGR5-KO mice (Fig. 1A and B).

**Fig. 8:**
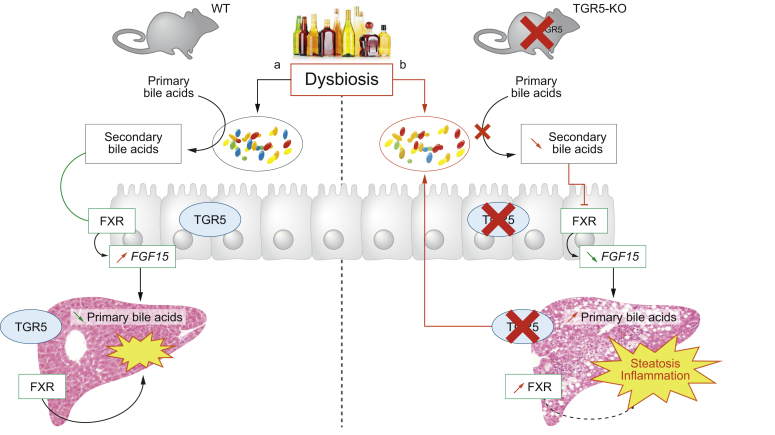
**Graphical summary of TGR5 and IM in alcohol-fed mice.** Total TGR5 deficiency in alcohol-fed mice induces dysbiosis of the intestinal microbiota, which is associated with a decrease in secondary BA levels in the plasma and liver. This decrease is associated with lower ileal FXR signaling and thus a decrease in FGF15 synthesis. Decreased FGF15 levels result in increased hepatic BA synthesis. The changes in the BA pool in the liver may result in liver FXR activation. These changes all result in an increase in liver injury. The solid lines are based on the results, whereas the dotted lines represent hypothetical relationships. BA, bile acids.

Overall, these results suggest that the lack of TGR5 in alcohol-fed mice is associated with specific dysbiosis, resulting in a profound decrease in secondary BA levels (Fig. 8). It is likely that such alteration of the composition of the BA pool affects the regulation of BA synthesis and MO recruitment, worsening liver steatosis, and inflammation.

## Discussion

We have previously shown that the IM has a causal role in individual susceptibility to ALD. We have also reported that the noxious IM associated with liver sensitivity to alcohol is associated with a modification of faecal BA composition, with elevated CDCA levels and low levels of UDCA.^3^^,^^35^ These results suggest that, along with alcohol intake, the IM can modify the BA pool, with a major impact on liver lesions. BAs, considered to be paracrine and endocrine molecules, act mainly through binding to the nuclear receptor FXR and the transmembrane receptor TGR5.^8^^,^^9^^,^^22^ It has been previously shown that ileal FXR activation is protective and that FXR deficiency has the opposite effect in alcohol-fed mice.[12], [13], [14] TGR5 is also reported to be protective in the setting of BA overload in mice,^21^ through yet incompletely defined mechanisms.^17^^,^^22^ However, the role of TGR5 in the context of alcohol consumption has been little explored.^23^ In a similar model of prolonged ethanol administration to mice, Iracheta-Vellve *et al.*^23^ explored the role of a TGR5 agonist and a combined TGR5 and FXR agonist and showed that TGR5 activation decreased hepatic steatosis, protected the mice from liver injury by modulating lipogenic gene expression, and decreased liver IL-1β levels. We also show, in the present study, that TGR5 protects against alcohol-induced liver steatosis and inflammation and highlight the role of the IM in these processes. Using a model of TGR5 deficiency, we show that the combination of TGR5 deficiency and alcohol intake results in IM dysbiosis, which markedly alters the composition of the BA pool, leading to dysregulation of both BA synthesis and inflammatory processes, converging towards the worsening of liver inflammation.

TGR5 deficiency worsens alcohol-induced liver injury relative to that of WT mice. We initially focused on liver MOs, as they play an important role in the initiation and progression of ALD, producing pro-inflammatory cytokines as a result of the translocation of bacterial products from the gut.^36^^,^^37^ In addition, TGR5 activation in monocytes/MOs, including liver MOs, dampens their pro-inflammatory profile.^18^ However, our data from isolated WT and TGR5-KO liver MOs of alcohol-fed mice suggest that, despite increased recruitment, liver inflammation in TGR5-KO mice is not associated with a pro-inflammatory phenotype of liver MOs. These data are similar to those reported for a mouse model of colitis, in which TGR5 deficiency enhanced the recruitment of classically activated MOs in the colonic lamina propria and worsened the severity of inflammation.^38^ BAs have been reported to possibly influence CCL-2 production by hepatocytes, suggesting that the differences in the BA profile observed in TGR5-KO mice may be involved in the higher production of CCL-2.^39^

As we previously showed that dysbiosis is associated with the severity of alcohol-induced liver injury, we compared the IM between the various groups of mice. The lack of TGR5 was associated with an altered IM and, importantly, this dysbiosis was aggravated by alcohol intake, with an increase in the abundance of the Gram-negative bacteria, Deferribacteres (including *Mucispirillum*) and *Alistipes*. IM transplantation experiments showed that the worsening of alcohol-induced liver injury and inflammation was, at least in part, related to the aggravation of dysbiosis in TGR5-KO mice. Comparisons of the IM between groups showed that only 3 taxa were specifically associated with the worsening of alcohol-induced liver injury, including *Mucispirillum* and *Allistipes.* Among *Alistipes*, it has been shown that the abundance of *A. putredinis* increases after alcohol administration,^40^ whereas an increase in the abundance of *Mucispirillum* was associated with liver injury in mice fed a high-fat diet.^41^^,^^42^ Overall, these data suggest that these bacteria may play a role in TGR5-associated worsening of alcohol-induced liver lesions.

Predicted metagenomic functions showed that the specific dysbiosis observed in alcohol-fed TGR5-KO mice was associated with a decrease in pathways involved in the transformation of primary BAs into secondary BAs, including cholylglycine hydrolase, which deconjugates BA in the intestine.^14^ Consistent with these data, the composition of the BA pool was strikingly modified in alcohol-fed TGR5-KO mice, with significantly lower plasma and liver secondary BA levels, mainly DCA, than in WT mice. Moreover, although the BA pool composition can be modified by the gut microbiota, BAs can also, in turn, modify the gut microbiota and contribute to dysbiosis.^43^ Therefore, the changes in the BA pool observed in the TGR5-KO mice may have contributed to the observed dysbiosis, and both BAs and the IM could be involved in the worsening of alcohol-induced liver injury. It is possible that these changes in the BA pool, including the decrease in DCA, which is an FXR agonist, may be responsible for the increase in BA synthesis in alcohol-fed TGR5-KO. Although, not statistically significant, our data also suggest that ileal FXR-dependent negative feedback of BA synthesis may also be dampened in the absence of TGR5, further deregulating the composition of the BA pool in these mice upon alcohol consumption.

In conclusion, TGR5 deficiency aggravates alcohol-induced liver lesions through the modulation of IM composition more than through exacerbation of the pro-inflammatory MO phenotype. The dysbiosis observed in the absence of TGR5 upon alcohol consumption markedly reshapes the composition of the BA pool and FXR-mediated adaptive responses, leading to more severe ALD. Our results open new avenues of investigation to determine whether we can improve ALD by targeting either TGR5 or intestinal bacteria.

## Financial support

This work was supported by INSERM, France; 10.13039/501100007486Université Paris-Sud, France; National French Society of Gastroenterology (SNFGE), France; Fondation pour la Recherche Médicale (FRM; DEQ20170336743), France; Association Française pour l’Etude du Foie (AFEF), France; Fondation pour la Recherche en Alcoologie (FRA), France; 10.13039/501100013945Institut de Recherches Internationales Servier (IRIS), France; Groupement Transversal INSERM sur le Microbiote (GPT microbiota), France. The group is a member of the Laboratory of Excellence 10.13039/501100011879LERMIT, supported by a grant from the 10.13039/501100001665Agence Nationale de la Recherche (ANR-10-LABX-33), France. MS received a doctoral scholarship from the Labex LERMIT.

## Authors’ contributions

Acquisition of data: MS, VP, GF, LW. Analysis of data: MS, AMC. Interpretation of data: AMC, TT, GM. Statistical analysis: MS, DC. Microbiota analysis: DC. Technical support: CH, NT. Bile-acid measurement and analysis: NGR, DR, LH. Histology, IHC, and quantifications: FMN, SD. Purchase of the TGR5 deficient mice: TT, GM. Study concept: GP, AMC. Study design: AMC. Study supervision: AMC. Drafting of the manuscript: MS, DC, TT, GM, AMC. Critical revision of the manuscript: GP. Obtaining funding: GP, AMC.

## Data availability

Data supporting the findings of this study are available from the corresponding author upon reasonable request.

## Conflicts of interest

During the last 3 years: AMC has received travel grants from Biocodex and royalties from Elsevier-Masson, John Libbey Eurotext, Solar, and Flammarion/Versilio; GP has received travel funds from Abbvie, Biocodex, Gilead, and MSD, consulting fees from Adare, Biocodex, Gilead, Pilèje, and Servier, and royalties from Elsevier-Masson, John Libbey Eurotext, Solar, and Flammarion/Versilio; DC has received travel grants from Biocodex and Gilead, and royalties from John Libbey Eurotext. All other authors declare no conflicts of interest.

Please refer to the accompanying ICMJE disclosure forms for further details.

## Supplementary data

Supplementary information.pdf

CTAT table.pdf
