# Supplementary material for: Bile acid-receptor TGR5 deficiency worsens liver injury in alcohol-fed mice by inducing intestinal microbiota dysbiosis
Source: JHEP Rep. 2021 Jan 19;3(2):100230. doi: 10.1016/j.jhepr.2021.100230 (PMC7903352; doi:10.1016/j.jhepr.2021.100230)
Supplement: Supplementary information.pdf [file mmc1.pdf]

Bile acid-receptor TGR5 deficiency worsens liver injury in alcohol-fed mice by inducing intestinal microbiota dysbiosis

Madeleine Spatz, Dragos Ciocan, Gregory Merlen, Dominique Rainteau, Lydie Humbert, Neuza Gomes-Rochette, Cindy Hugot, Nicolas Trainel, Françoise Mercier-Nomé, Séverine Domenichini, Virginie Puchois, Laura Wrzosek, Gladys Ferrere, Thierry Tordjmann, Gabriel Perlemuter, Anne-Marie Cassard

Table of contents

Fig. S1...2

Fig. S2...3

Fig. S3...4

Fig. S4...5

Fig. S5...6

Table S1...7

Fig. S1

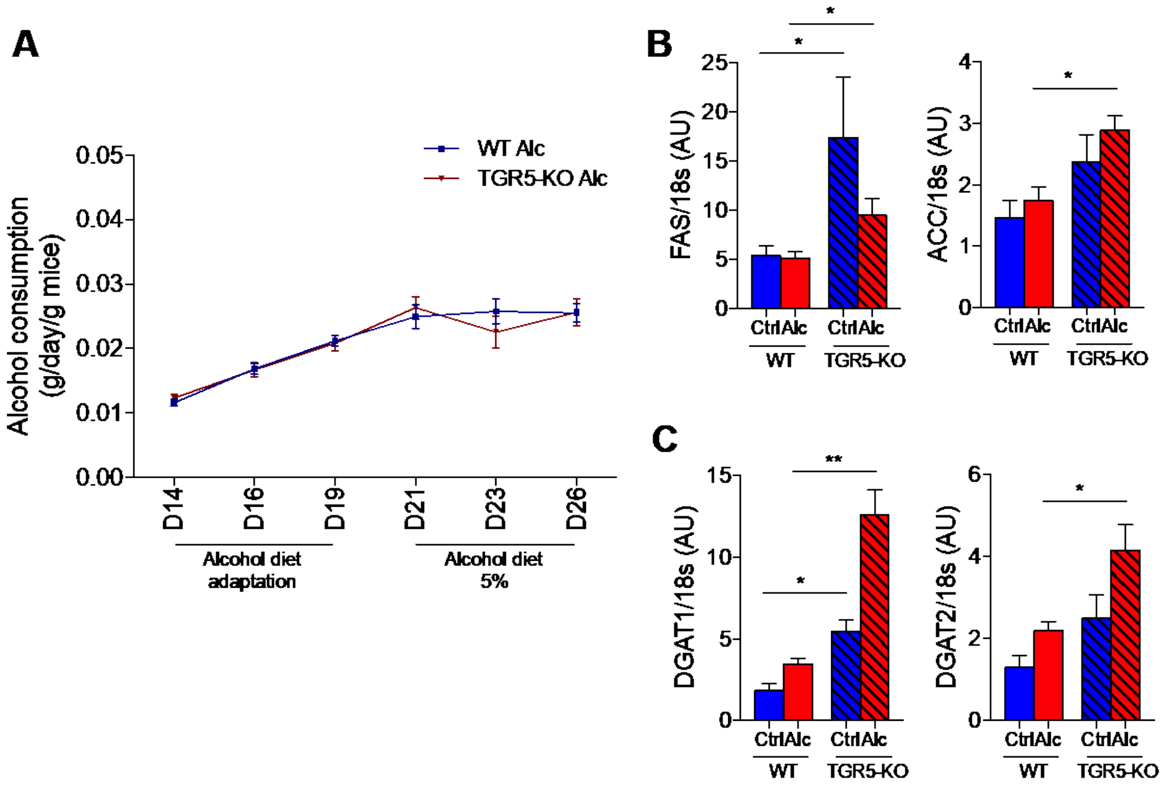

Fig. S1. Alcohol consumption and quantification of liver mRNA expression involved in lipid metabolism. Wildtype (WT) and TGR5-deficient mice (TGR5-KO) mice were fed alcohol (Alc) or isocaloric maltodextrin (Ctrl). (A) Similar alcohol consumption in alcohol fed WT and TGR5-deficient mice. (B-C) Liver mRNA expression of genes quantified by qPCR related to (B) lipogenesis and (C) triglyceride synthesis. Dunn's test, $*p < 0.05$, $**p < 0.01$. WT Ctrl n=4; WT Alc n=6; TGR5-KO Ctrl n=4; TGR5-KO Alc n=6.

Fig. S2

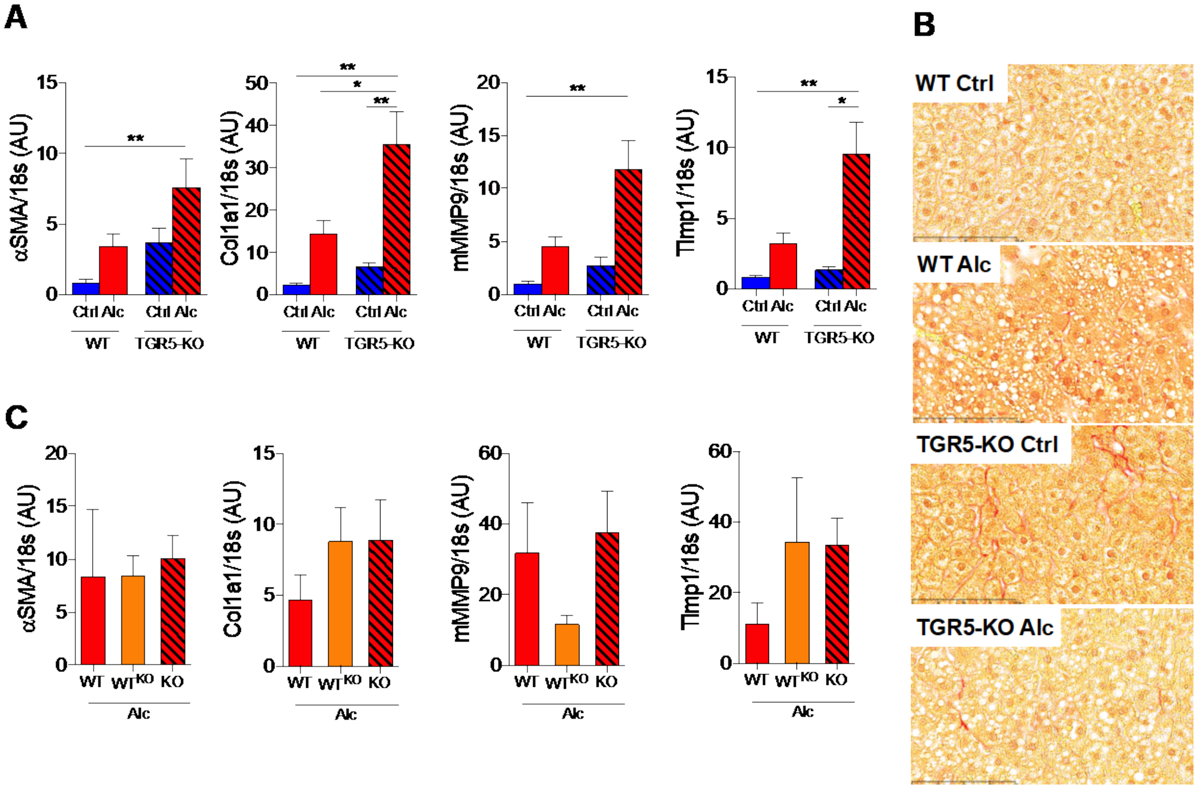

Fig. S2. Liver fibrosis. (A-B) WT and TGR5-KO (KO) mice were fed alcohol (Alc) or isocaloric maltodextrin (Ctrl). (C) WT, KO and WT mice transplanted with the IM of KO mice (WT^{KO}) were fed alcohol. (A and C) Liver mRNA expression of genes involved in fibrosis. (B) Representative pictures of picro-sirius red staining showing fibrotic scars in the liver (scale bar=100µm). Dunn's test, $*p < 0.05$, $**p < 0.01$. WT Ctrl n=4; WT Alc n=6; TGR5-KO; Ctrl n=4; TGR5-KO Alc n=6; WT n=5; WT^{KO} n=5; KO n=9.

Fig. S3

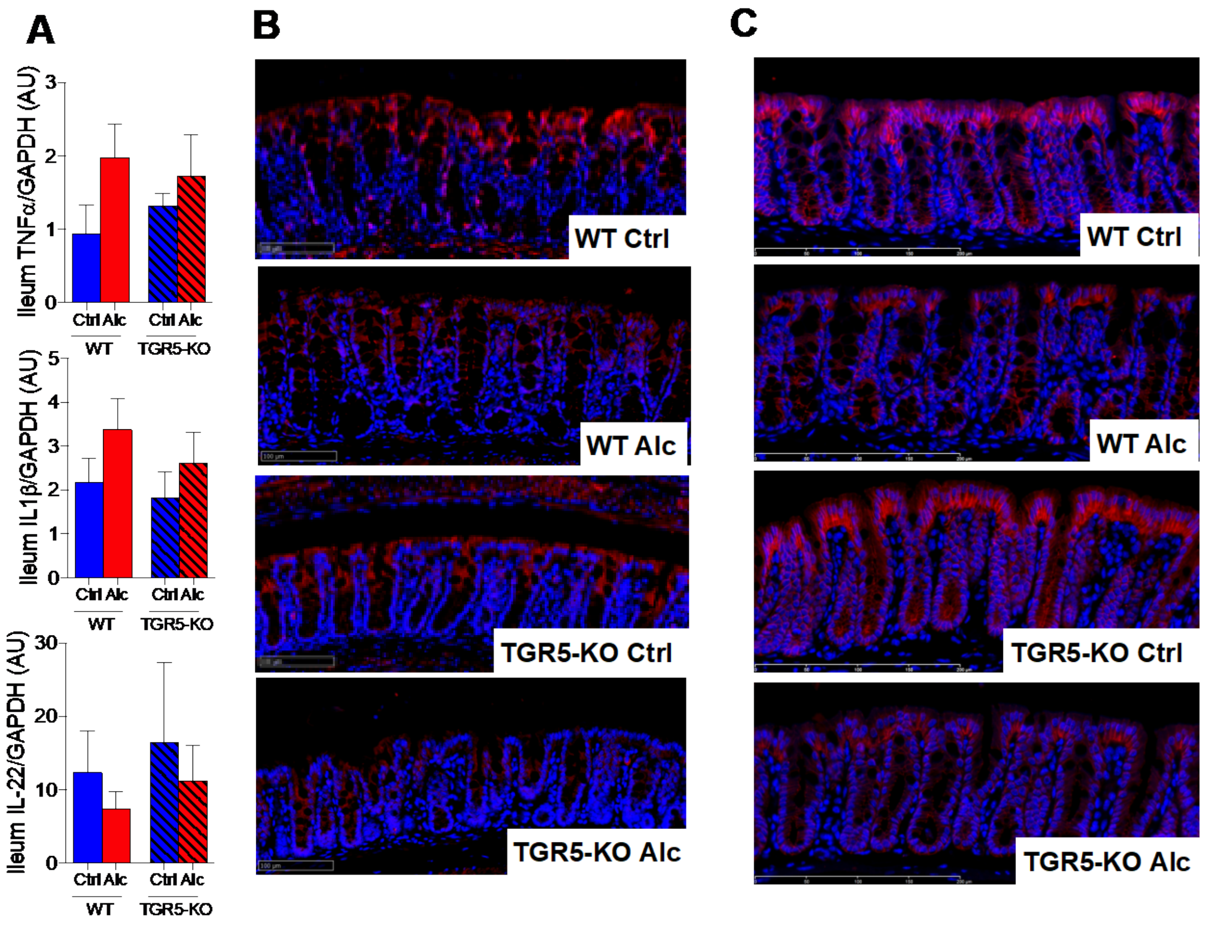

Fig. S3. Intestinal fitness. WT and TGR5-KO mice were fed alcohol (Alc) or isocaloric maltodextrin (Ctrl). (A) Ileum mRNA expression of genes involved in inflammation (Dunn's test). (B) Immunofluorescence of ZO-1 in the colon (scale bar = 100µm). (C) Immunofluorescence of Occludine in the colon (scale bar = 200µm). WT Ctrl n=4; WT Alc n=6; TGR5-KO Ctrl n=4; TGR5-KO Alc n=6.

Fig. S4

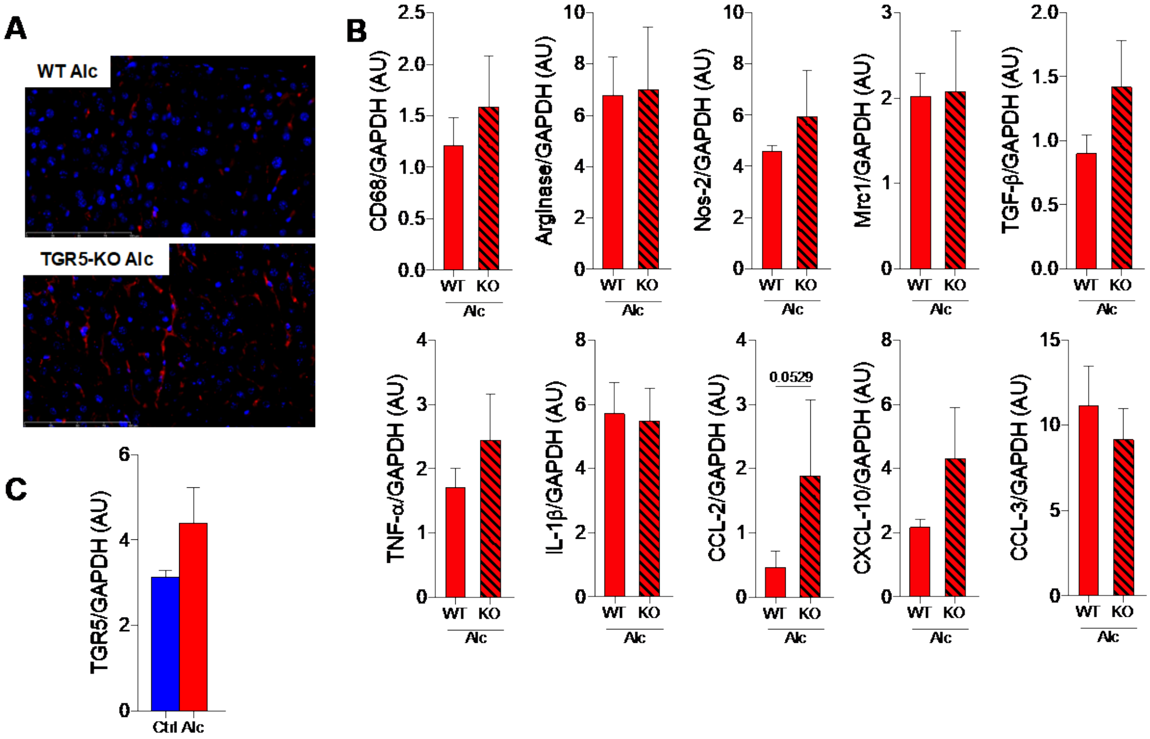

Fig. S4. mRNA expression in isolated liver macrophages from WT and TGR5-deficient alcohol-fed mice. WT and TGR5-KO (KO) mice were fed alcohol. (A) Immunohistochemistry of a liver section with F4/80 antibody and DAPI (scale bar=100µm). (B-C) mRNA expression of genes related to (B) the inflammatory phenotype in liver macrophages and (C) TGR5 in the liver of WT mice. Mann-Whitney test, WT n=12; KO n=6; Ctrl n=7; Alc n=9

Fig. S5

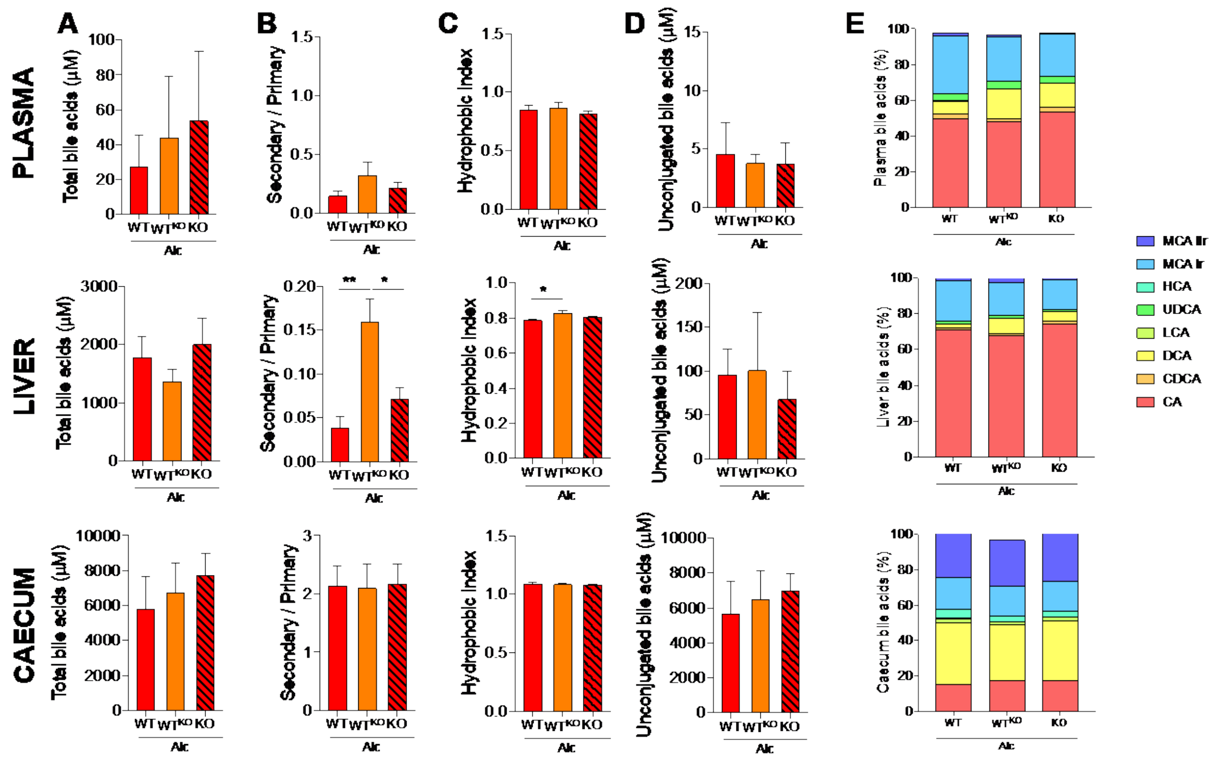

Fig. S5. Bile acids in alcohol-fed WT, KO and WT^{KO} mice. WT, KO, and WT^{KO} were fed alcohol. (A-E) Bile-acid composition in the plasma (upper), in the liver (middle) and in the caecum (lower). (A) Total bile acids. (B) Ratio of Secondary/Primary bile acids. (C) Hydrophobic index. (D) Unconjugated bile acids. (E) Percentage of each bile acid. Dunn's test, $*p < 0.05$, $**p < 0.01$. WT n=5; WT^{KO} n=5; KO n=9.

Table S1. Oligonucleotides used in this study.

Name	5'- Forward - 3'	5'- Reverse - 3'
18s	5'-GTA-ACC-CGT-TGA-ACC-CCA-TT-3'	5'-CCA-TCC-AAT-CGG-TAG-TAG-CG-3'
ACC	5'-AGC-AGA-TCC-GCA-GCT-TG-3'	5'-ACC-TCT-GCT-CGC-TGA-GTG-C-3'
alpha-SMA	5'-CCT-GGT-GTG-CGA-CAA-TG-3'	5'-TGC-TCT-GGG-CTT-CAT-CC-3'
Arginase	5'-CTC-CAA-GCC-AAA-GTC-CTT-AGA-G 3'	5'-AGG-AGC-TGT-CAT-TAG-GGA-CAT-C-3'
CCL-2	5' AGG-TCC-CTG-TCA-TGC-TTC-TG-3'	5'-TCT-GGA-CCC-ATT-CCT-TCT-TG-3'
CCL-20 (Mm_Ccl20_1_SG)	Ref Qiagen : QT00261898	
CCL-3 (Mm_Ccl3_1_SG)	Ref Qiagen : QT00248199	
CCL-5	5'-CAC-CTG-CCT-CAC-CAT-ATG-GC-3'	5'-GGC-GGT-TCC-TTC-GAG-TGA-CA-3'
CD68	5'-CTT-CCC-ACA-GGC-AGC-ACA-G-3'	5'-AAT-GAT-GAG-AGG-CAG-CAA-GAG-G-3'
Col1a1	5'- ACT-GCA-ACA-TGG-AGA-CAG-GTC-AGA-3'	5'-ATC-GGT-CAT-GCT-CTC-TCC-AAA-CCA-3'
CXCL-10 (Mm_Cxcl10_1_SG)	Ref Qiagen : QT00093436	
Cyp27a1 (Mm_Cyp27a1_1_SG)	Ref Qiagen : QT00155778	
Cyp7a1 (Mm_Cyp7a1_1_SG)	Ref Qiagen : QT00121569	
Cyp8b1 (Mm_Cyp8b1_1_SG)	Ref Qiagen : QT00249907	
DGAT1	5'-TTC-CGC-CTC-TGG-GCA-TT-3'	5'-AGA-ATC-GGC-CCA-CAA-TCC-A-3'
DGAT2	5'-AGT-GGC-AAT-GCT-ATC-ATC-CGT-GT-3'	5'-AAG-GAA-TAA-GTG-GGA-ACC-CAG-ATC-A-3'
F4/80	5'-CTT-TGG-CTA-TGG-GCT-TCC-AGT-C-3'	5'-GCA-AGG-AGG-ACA-GAG-TTT-ATC-GTC-3'
FAS	5'-TTC-CAA-GAC-GAA-AAT-GAT-GC-3'	5'-AAT-TGT-GGG-ATC-AGG-AGA-GC-3'

FGF15	5'-GAG-GAC-CAA-AAC-GAA-CGA-AAT-T-3'	5'-ACG-TCC-TTG-ATG-GCA-ATC-G-3’
FXR	5'-CCA-ACC-TGG-GCT-TCT-ACC-C-3'	5'-CAC-ACA-GCT-CAT-CCC-CTT-T-3’
GAPDH	5'-GTG-GAC-CTC-ATG-GCC-TAC-AT-3'	5'-TGT-GAG-GGA-GAT-GCT-CAG-TG-3'
IL-1beta	5'-AAG-GTC-CAC-GGG-AAA-GAC-AC-3'	5'-AGC-TTC-AGG-CAG-GCA-GTA-TC-3'
IL-22	5'-TTT-AAC-TCC-CTT-GGC-GCA-AAA-3'	5'-CTT-TCC-CTC-CGC-ATT-GAC-AC-3'
mMMP9	5’-GTC-CAG-ACC-AAG-GGT-ACA-GC-3’	5’-ATA-CAG-CGG-GTA-CAT-GAG-CG-3’
Mrc1	5'-GGA-CGA-GCA-GGT-GCA-GTT-3'	5'-CAA-CAC-ATC-CCG-CCT-TTC-3'
Muc2	5'-CCC-AGA-AGG-GAC-TGT-GTA-TG-3'	5'-TTG-TGT-TCG-CTC-TTG-GTC-AG-3'
Nos-2	5’-CCA-AGC-CCT-CAC-CTA-CTT-CC-3’	5’-CTC-TGA-GGG-CTG-ACA-CAA-GG-3’
SREBP	5'- AAC-GTC-ACT-TCC-AGC-TAG-AC -3’	5'- CCA-CTA-AGG-TGC-CTA-C AG-AGC -3’
TGF-beta	5'-GCA-ACA-TGT-GGA-ACT-CTA-CCA-GAA-3'	5'-GAC-GTC-AAA-AGA-CAG-CCA-CTC-A3'
TGR5	5'-GTC-AGC-TCC-CTG-TTC-TTT-GC-3'	5'-CAG-GAG-GCC-ATA-AAC-TTC-CA-3'
Timp1	Ref Qiagen : QT00996282	
TNF-alpha	5'-TGG-GAG-TAG-ACA-AGG-TAC-AAC-CC-3'	5'-CAT-CTT-CTC-AAA-ATT-CGA-GTG-ACA-A-3'
