# Supplementary material for: Bile acid-receptor TGR5 deficiency worsens liver injury in alcohol-fed mice by inducing intestinal microbiota dysbiosis
Source: JHEP Rep. 2021 Jan 19;3(2):100230. doi: 10.1016/j.jhepr.2021.100230 (PMC7903352; doi:10.1016/j.jhepr.2021.100230)
Supplement: CTAT table.pdf [file mmc2.pdf]

JHEP Reports

CTAT methods

Tables for a "Complete, Transparent, Accurate and Timely account" (CTAT) are now mandatory for all revised submissions. The aim is to enhance the reproducibility of methods.

- Only include the parts relevant to your study
- Refer to the CTAT in the main text as 'Supplementary CTAT Table'
- Do not add subheadings
- Add as many rows as needed to include all information
- Only include one item per row

If the CTAT form is not relevant to your study, please outline the reasons why:

1.1 Antibodies

Name	Citation	Supplier	Cat no.	Clone no.
F4/80 (anti-rat)		Bio-Rad, France	MCA497GA	CI:A3-I
Alexa Fluor 594-anti rat		Thermo Fisher, France	A21209	polyclonal
Streptavidin-horseradish peroxidase complex		LSAB kit, Dako	K5001	N/A
ZO-1 (anti-rabbit)		Abcam, UK	ab96587	R40-76
Occludine (anti-rabbit)		Abcam, UK	ab216327	F-11
Alexa Fluor 594 anti-rabbit		Thermo Fisher Scientific	A32740	polyclonal
Hoechst		Molecular Probes	H-3570	N/A

1.2 Cell lines

Name	Citation	Supplier	Cat no.	Passage no.	Authentication test method

1.3 Organisms

Name	Citation	Supplier	Strain	Sex	Age	Overall n number
TGR5-KO		T. Tordjmann	Mice	Female	Seven- or ten-week-old	25

Wild type		T. Tordjmann	Mice	Female	Seven- or ten-week-old	32
C57BL/6J		Janvier, France	Mice	Female	Seven-week-old	34

1.4 Sequence based reagents

Name	Sequence	Supplier
18s-F	5'-GTA-ACC-CGT-TGA-ACC-CCA-TT-3'	Eurogentec
18s-R	5'-CCA-TCC-AAT-CGG-TAG-TAG-CG-3'	Eurogentec
ACC-F	5'-AGC-AGA-TCC-GCA-GCT-TG-3'	Eurogentec
ACC-R	5'-ACC-TCT-GCT-CGC-TGA-GTG-C-3'	Eurogentec
α-SMA-F	5'-CCT-GGT-GTG-CGA-CAA-TG-3'	Eurogentec
α-SMA-R	5'-TGC-TCT-GGG-CTT-CAT-CC-3'	Eurogentec
Arginase-F	5'-CTC-CAA-GCC-AAA-GTC-CTT-AGA-G 3'	Eurogentec
Arginase-R	5'-AGG-AGC-TGT-CAT-TAG-GGA-CAT-C-3'	Eurogentec
CCL-2-F	5' AGG-TCC-CTG-TCA-TGC-TTC-TG-3'	Eurogentec
CCL-2-R	5'-TCT-GGA-CCC-ATT-CCT-TCT-TG-3'	Eurogentec
CCL-20 (Mm_Ccl20_1_SG)	QT00261898	Qiagen
CCL-3 (Mm_Ccl3_1_SG)	QT00248199	Qiagen
CCL-5-F	5'-CAC-CTG-CCT-CAC-CAT-ATG-GC-3'	Eurogentec
CCL-5-R	5'-GGC-GGT-TCC-TTC-GAG-TGA-CA-3'	Eurogentec
CD68-F	5'-CTT-CCC-ACA-GGC-AGC-ACA-G-3'	Eurogentec
CD68-R	5'-AAT-GAT-GAG-AGG-CAG-CAA-GAG-G-3'	Eurogentec
Col1a1-F	5'- ACT-GCA-ACA-TGG-AGA-CAG-GTC-AGA-3'	Eurogentec
Col1a1-R	5'-ATC-GGT-CAT-GCT-CTC-TCC-AAA-CCA-3'	Eurogentec
CXCL-10 (Mm_Cxcl10_1_SG)	QT00093436	Qiagen
Cyp27a1 (Mm_Cyp27a1_1_SG)	QT00155778	Qiagen
Cyp7a1 (Mm_Cyp7a1_1_SG)	QT00121569	Qiagen
Cyp8b1 (Mm_Cyp8b1_1_SG)	QT00249907	Qiagen
DGAT1-F	5'-TTC-CGC-CTC-TGG-GCA-TT-3'	Eurogentec
DGAT1-R	5'-AGA-ATC-GGC-CCA-CAA-TCC-A-3'	Eurogentec
DGAT2-F	5'-AGT-GGC-AAT-GCT-ATC-ATC-CGT-GT-3'	Eurogentec
DGAT2-R	5'-AAG-GAA-TAA-GTG-GGA-ACC-CAG-ATC-A-3'	Eurogentec
F4/80-F	5'-CTT-TGG-CTA-TGG-GCT-TCC-AGT-C-3'	Eurogentec
F4/80-R	5'-GCA-AGG-AGG-ACA-GAG-TTT-ATC-GTC-3'	Eurogentec
FAS-F	5'-TTC-CAA-GAC-GAA-AAT-GAT-GC-3'	Eurogentec
FAS-R	5'-AAT-TGT-GGG-ATC-AGG-AGA-GC-3'	Eurogentec
FGF15-F	5'-GAG-GAC-CAA-AAC-GAA-CGA-AAT-T-3'	Eurogentec
FGF15-R	5'-ACG-TCC-TTG-ATG-GCA-ATC-G-3'	Eurogentec
FXR-F	5'-CCA-ACC-TGG-GCT-TCT-ACC-C-3'	Eurogentec
FXR-R	5'-CAC-ACA-GCT-CAT-CCC-CTT-T-3'	Eurogentec
GAPDH-F	5'-GTG-GAC-CTC-ATG-GCC-TAC-AT-3'	Eurogentec
GAPDH-R	5'-TGT-GAG-GGA-GAT-GCT-CAG-TG-3'	Eurogentec
IL-1β-F	5'-AAG-GTC-CAC-GGG-AAA-GAC-AC-3'	Eurogentec
IL-1β-R	5'-AGC-TTC-AGG-CAG-GCA-GTA-TC-3'	Eurogentec

IL-22-F	5'-TTT-AAC-TCC-CTT-GGC-GCA-AAA-3'	Eurogentec
IL-22-R	5'-CTT-TCC-CTC-CGC-ATT-GAC-AC-3'	Eurogentec
mMMP9-F	5'-GTC-CAG-ACC-AAG-GGT-ACA-GC-3'	Eurogentec
mMMP9-R	5'-ATA-CAG-CGG-GTA-CAT-GAG-CG-3'	Eurogentec
Mrc1-F	5'-GGA-CGA-GCA-GGT-GCA-GTT-3'	Eurogentec
Mrc1-R	5'-CAA-CAC-ATC-CCG-CCT-TTC-3'	Eurogentec
Muc2-F	5'-CCC-AGA-AGG-GAC-TGT-GTA-TG-3'	Eurogentec
Muc2-R	5'-TTG-TGT-TCG-CTC-TTG-GTC-AG-3'	Eurogentec
Nos-2-F	5'-CCA-AGC-CCT-CAC-CTA-CTT-CC-3'	Eurogentec
Nos-2-R	5'-CTC-TGA-GGG-CTG-ACA-CAA-GG-3'	Eurogentec
SREBP1-F	5'- AAC-GTC-ACT-TCC-AGC-TAG-AC -3'	Eurogentec
SREPB1-R	5'- CCA-CTA-AGG-TGC-CTA-C AG-AGC -3'	Eurogentec
TGF-β-F	5'-GCA-ACA-TGT-GGA-ACT-CTA-CCA-GAA-3'	Eurogentec
TGF-β-R	5'-GAC-GTC-AAA-AGA-CAG-CCA-CTC-A3'	Eurogentec
TGR5-F	5'-GTC-AGC-TCC-CTG-TTC-TTT-GC-3'	Eurogentec
TGR5-R	5'-CAG-GAG-GCC-ATA-AAC-TTC-CA-3'	Eurogentec
Timp1	QT00996282	Qiagen
TNF-α-F	5'-TGG-GAG-TAG-ACA-AGG-TAC-AAC-CC-3'	Eurogentec
TNF-α-R	5'-CAT-CTT-CTC-AAA-ATT-CGA-GTG-ACA-A-3'	Eurogentec

1.5 Biological samples

Description	Source	Identifier

1.6 Deposited data

Name of repository	Identifier	Link

1.7 Software

Software name	Manufacturer	Version
ImageJ	U. S. National Institutes of Health, Bethesda, Maryland, USA	https://imageJ.nih.gov
NDP.view2	Hamamatsu, Japan	2
R	Foundation for Statistical Computing, Vienna, Austria	2.14.1
LEfSe	The Huttenhower Lab, Harvard, USA	http://huttenhower.sph.harvard.edu/galaxy/
PICRUSt	The Huttenhower Lab, Harvard, USA	http://huttenhower.sph.harvard.edu/galaxy/
Graphpad Prism	Graphpad Software Inc, La Jolla, California, USA	7.0a

1.8 Other (e.g. drugs, proteins, vectors etc.)

1.9 Please provide the details of the corresponding methods author for the manuscript:

Anne-Marie Cassard, INSERM U996, 32 rue des Carnets, Clamart, F-92140, France. E-mail: cassard.doulcier@u-psud.fr

2.0 Please confirm for randomised controlled trials all versions of the clinical protocol are included in the submission. These will be published online as supplementary information.
